# Roles and Functions of Exosomal Non-coding RNAs in Vascular Aging

**DOI:** 10.14336/AD.2019.0402

**Published:** 2020-02-01

**Authors:** Yu-Qing Ni, Xiao Lin, Jun-Kun Zhan, You-Shuo Liu

**Affiliations:** Department of Geriatrics, Institute of Aging and Geriatrics, the Second Xiangya Hospital, Central South University, Changsha, Hunan, 410011, China

**Keywords:** exosomes, endothelial cells, vascular smooth muscle cells, vascular aging, vascular diseases

## Abstract

Aging is a progressive loss of physiological integrity and functionality process which increases susceptibility and mortality to diseases. Vascular aging is a specific type of organic aging. The structure and function changes of endothelial cells (ECs) and vascular smooth muscle cells (VSMCs) are the main cause of vascular aging, which could influence the threshold, process, and severity of vascular related diseases. Accumulating evidences demonstrate that exosomes serve as novel intercellular information communicator between cell to cell by delivering variety biologically active cargos, especially exosomal non-coding RNAs (ncRNAs), which are associated with most of aging-related biological and functional disorders. In this review, we will summerize the emerging roles and mechanisms of exosomal ncRNAs in vascular aging and vascular aging related diseases, focusing on the role of exosomal miRNAs and lncRNAs in regulating the functions of ECs and VSMCs. Moreover, the relationship between the ECs and VSMCs linked by exosomes, the potential diagnostic and therapeutic application of exosomes in vascular aging and the clinical evaluation and treatment of vascular aging and vascular aging related diseases will also be discussed.

Review

## 1.Introduction

Vascular aging is a specific type of organic aging, leading to major adverse cardiovascular events including restenosis, atherosclerosis, vascular calcification (VC) and pulmonary hypertension. It could influence the threshold, process, and severity of various cardiovascular diseases. Vascular aging occurs mainly in the inner and medial layers of vessel wall [1]. Therefore, the dysfunctions of endothelial cells (ECs) and vascular smooth muscle cells (VSMCs) including cellular apoptosis, senescence, proliferation, inflammation, and the phenotypic change of VSMCs play an important role in vascular aging [2]. In terms of the cells,ECs and VSMCs may undergo both replicative and induced senescence in vivo. At the level of vascular tissue, progenitor cells may be involved in damage and replacement of senescent cells. If progenitor cells are depleted, and ECs and VSMCs are less self-renewing, it may lead to vascular aging. In terms of function, it is featured by increased stiffness, reduced sensitivity to vasodilators, increased sensitivity to vasoconstrictors and decreased angiogenic capacity.

Exosomes are endosomal origin from multivesicular bodies with the smallest diameter of 30-100nm. Exosomes derived from different cells transport distinct proteins, lipids, nucleic acids such as mRNA, microRNAs (miRNAs) and long noncoding RNAs (lncRNAs) [3, 4]. Because of the variety and abundance of specific cargos, exosomes keep a great potential application for diagnostic and prognostic biomarkers and therapeutic tools. Exosomes, exploited as gene regulators and information communicators, contributed to many essential physiological and pathophysiological processes including proliferation, differentiation, apoptosis, homeostasis, and migration [5, 6]. Accordingly, increasing evidence has demonstrated that aging-related biological and functional disorders are associated with alterations in exosomes, suggesting that exosomes are potential regulators for vascular aging [7-10].

**Table 1:** Exosomal non-coding RNAs implicated in ECs functions.

Exosomal non-coding RNAs	Cargos	Targets	ECs functions	Reference
Exosomal miRNAs	miR-122-5p	HGF	pro-proliferation, pro-migration	[23]
	miR-210-3p	HGF	pro-proliferation, pro-migration	[23]
	miR-296-5p	HGF	pro-proliferation, pro-migration	[23]
	miR-376c-3p	HGF	pro-proliferation, pro-migration	[23]
	miR-214	ATM	pro-proliferation, anti-senescence, pro-angiogenesis	[24]
	miR-17	ANGPT1 STAT3	pro-proliferation, pro-migration, anti-inflammation	[25] [35]
	miR-19	ANGPT1	pro-proliferation, pro-migration	[25]
	miR-20a	ANGPT1	pro-proliferation, pro-migration	[25]
	miR-30c	ANGPT1	pro-proliferation, pro-migration	[25]
	miR-126	ANGPT1	pro-proliferation, pro-migration	[25]
	miR-92a	SOCS5	anti- proliferation, pro-inflammation	[26] [30]
	miR-21	RhoB	anti- proliferation, pro-inflammation	[27] [31]
	miR-24	Sp1	anti- proliferation	[28]
	miR-15a	VEGF, NF-kB	pro-inflammation	[32, 33]
	miR-27a	VEGF, EGFR	pro-inflammation	[32, 33]
	miR-34a	BCL2, SIRT1	pro-inflammation	[32, 33]
	miR-223	ICAM-1	anti-inflammation	[34]
	miR-125a	DLL4	pro-angiogenesis	[36]
	miR-181b-5p	TRPM7	pro-angiogenesis	[37]
	miR-106b-5p	ANG2	anti- angiogenesis	[38]
	miR-320	IGF-1, Hsp20, Ets2	anti- angiogenesis	[39]
Exosomal lncRNAs	HOTTIP	cyclin D1, PCNA	pro-proliferation, pro-migration	[48]
	POU3F3	bFGF, VEGF	pro-proliferation, pro-migration, pro-angiogenesis	[49]
	MALAT1	IL-6, TNF-α, SAA3	anti- proliferation, pro-migration, pro-inflammation	[50] [59, 60]
	HOTAIR	VEGF	pro-angiogenesis	[53]
	H19	Unknown	pro-angiogenesis	[54]
	CCAT2	TGFβ, Bcl-2	pro-angiogenesis, anti-apoptosis	[55]
	Meg3	Unknown	anti- angiogenesis	[57]
	GAS5	P53, Caspase 3, Caspase 7	pro-apoptosis	[58]

HGF: hepatocyte growth factor; ATM: ataxia telangiectasia mutated; ANGPT1: angiopoietin-1; STAT3: signal transducer and activator of transcription 3; SOCS5: suppressor of cytokine signaling 5; RhoB: ras homologue family member B; Sp1: specificity protein 1; VEGF: vascular endothelial growth factor; NF-kB: nuclear factor-kappa B; EGFR epidermal growth factor receptor; BCL2: B-cell CLL/lymphoma 2; SIRT1: silent information regulator 1; ICAM-1: intercellular adhesion molecule-1; DLL4: delta-like 4; TRPM7: transient receptor potential melastatin 7; ANG2: Angiopoietin 2; IGF-1: insulin-like growth factor-1; Hsp20: small heat-shock protein 20; Ets2: E26 transformation-specific 2; PCNA: proliferating cell nuclear antigen; bFGF: basic fibroblast growth factor; IL-6: interleukin 6; TNF-α: tumor necrosis factor alpha; SAA3: serum amyloid antigen 3;TGFβ:transforming growth factor β.

Non-coding RNAs (ncRNAs) are mainly including miRNAs, lncRNAs as well as circular RNAs(cirRNAs) [11]. Recently, several ncRNAs have emerged as important molecules regulating the complexity of vascular aging. A large number of studies dealing with circulating exosomes and their cargos prove that exosomal miRNAs and lncRNAs were involved in regulating ECs and VSMCs dysfunction, which significantly affecting the process of vascular aging [12, 13]. However, there are fewer researches to explore the role of cirRNAs incorporated into exosomes in regulating vascular aging. Therefore, in this review, we summarize the current knowledge about the exosomal ncRNAs focusing on the role of miRNAs and lncRNAs in vascular aging, especially targeting at the effects on ECs and VSMCs and the information communication between these cells.

## 2.The role of exosomes in vascular aging

Vascular aging is tightly associated to alterations in the physiological functions and structural properties of the vascular wall, mainly in ECs and VSMCs. Emerging evidence suggest that exosomes acted as natural vehicles for the delivery of nucleic acids and signaling molecules [14, 15]. The important role of exosomes in modulating vascular aging is exploited with growing interests in exosomal cargo studies, especially the exosomal miRNAs and lncRNAs. Hence, better understanding of vascular biological and functional changes with aging is vital to against vascular aging related diseases.

### 2.1 Exosomal ncRNAs and ECs functions

ECs, the monolayer lining blood vessels, regulate the vascular integrity and homeostasis [16, 17]. An extensive surge of abnormalities of ECs, collectively termed “endothelial dysfunction”, are fundamental contributors to the pathogenesis of vascular diseases. When the endothelium was exposed to micro-environmental stimuli, such as oxidative stress, hypoxia, inflammatory mediators, hypertension, hyperglycemia, or shear stress, the ECs would undergo alterations including proliferation, apoptosis, migration, senescence, angiogenesis, inflammation, and functional networks which eventually affect vascular aging [18]. In this part, we focused on the relationship between specific contents of exosomal ncRNAs and ECs functions (Table 1).

#### 2.1.1 Exosomal miRNAs and ECs functions

One of striking aspects of the content of exosomes is that they are highly enriched in miRNAs that could target the 3’untranslated region (3’UTR) of specific mRNAs to inhibit, in most cases, their translation. Numerous lines of evidence supported that exosomal miRNAs were involved in regulating ECs proliferation,apoptosis, senescence, angiogenesis, and inflammation [19-21].

ECs proliferation is vital to promote endothelial healing. Accumulating evidence had indicated that exosomal miRNAs acted as critical regulators to participate in EC proliferation [22, 23]. It had been reported that exosomes carried miR-122-5p, miR-210-3p, miR-296-5p, and miR-376c-3p were significantly increased in human umbilical endothelia cell (HUVEC) and may account for the different regulation of ECs proliferation and migration [23]. Besides, miR-214 was incorporated into exosomes, leading to ECs proliferation and angiogenesis both *in vitro* and *in vivo* [24]. Garcia et al. proved that exosomes containing an enrichment of proangiogenic miR-17, miR-19, miR-20a, miR-30c and miR-126 from cardiomyocytes could promote the proliferation and migration of ECs [25]. On the contrary, a large number of researches reported that miRNAs had an effect of inhibiting the proliferation of ECs, including miR-92a [26], miR-21 [27] and miR-24 [28]. It had been shown that miR-92a could modulate KLF4 and MKK4 expression in ECs and inhibition of miR-92a might increase ECs proliferation and migration [26].

ECs inflammation induced by stimuli such as shear stress, inflammatory cytokines, or hypoxia, is more susceptible to cardiovascular diseases. Recent reports had shown that several exosomal miRNAs were involved in the regulatory mechanisms of cellular inflammation by controlling leukocyte activation and infiltration through the vascular wall [29]. As the proinflammatory regulator in ECs, miR-92a could activate inflammatory cytokines and chemokines and promote monocyte adhesion [30] while miR-21 could induce the expression of C-C motif chemokine 2 (CCL2) and vascular cell adhesion protein 1 (VCAM-1) by enhancing the activity of the transcription factor AP-1 [31]. It had been demonstrated that some miRNAs from exosomes (miR-15a, miR-27a and miR-34a) were increased in patients with sepsis, which could modulate the inflammatory response [32, 33]. Oppositively, Li et al. discovered that exosomes containing miR-223 inhibited intercellular adhesion molecule-1 (ICAM-1) expression during inflammation through regulating the NF-κB and MAPK pathways [34]. It had been confirmed that exosomes derived from mesenchymal stem cells contained miR-17 superfamily that played an anti-inflammatory role in pulmonary ECs through the suppression of signal transducer and activator of transcription 3 (STAT3) [35].

ECs angiogenesis is responsible for the progression of vascular aging, and emerging evidence suggest that exosomal miRNAs are signaling molecules that modulate the microenvironment and promote angiogenesis of ECs [36]. Several miRNAs are responsible for angiogenesis while others are the so-called antiangiogenic miRNAs. Liang et al. indicated that exosomes could transfer miR-125a to ECs and promote angiogenesis by repressing angiogenic inhibitor delta-like 4 (DLL4) [36]. Yang et al. showed that exosomes could promote the ECs angiogenesis induced by oxygen-glucose deprivation via miR-181b-5p/TRPM7 axis [37]. On the contrary, it had been proven that up-regulation of exosomal miR-106b-5p suppressed angiogenesis in ECs by overexpression of angiopoietin 2 [38]. Besides, it had been indicated that exosomes exerted an anti-angiogenic function through transfer of miR-320 into ECs [39].

ECs apoptosis and senescence results in a disruption of the endothelium barrier and creating leaks that destroy the vascular wall integrity, which contribute to the development of atherosclerosis [40]. Exosomes containing miR-214 repressed the expression of ataxia telangiectasia mutated in recipient cells, thereby preventing senescence and inducing angiogenesis and migration [24].

#### 2.1.2 Exosomal lncRNAs and ECs function

LncRNAs contains a class of transcripts longer than 200 nucleotides [41]. They regulate gene expression at transcription, epigenetic, and translation levels, coordinating and integrating multiple signaling pathways involved in cellular proliferation, differentiation, and homeostasis [42, 43]. Several lines of evidence had demonstrated that lncRNAs were involved in different aspects during the process of vascular aging including cellular differentiation, proliferation, apoptosis, and inflammation [44-47]..

Recent report showed that exosomal lncRNA HOTTIP promoted ECs proliferation and migration via activation of the Wnt/β-catenin pathway [48]. Another study demonstrated that exosomal lncRNA POU3F3 could increase ECs proliferation and migration [49]. Besides, it had been reported that silencing of exosomal lncRNA MALAT1 by GapmeRs or small interfering RNAs induced a switch of the ECs phenotype to a promigratory but antiproliferative state that resulted in impaired ECs proliferation and reduced vessel growth [50].

A large number of studies had reported that several lncRNAs played an important role in regulating angiogenesis of ECs [51, 52]. For instance, the lncRNA HOTAIR was packed into exosomes secreted by glioma cells and conveyed to ECs. Then, HOTAIR stimulated angiogenesis by increasing the expression of VEGFA [53]. In addition, it had been reported that CD90^+^ cells modulated ECs angiogenesis through the release of exosomes containing lncRNA H19 by upregulating VEGF production [54]. Besides, exosomal lncRNA POU3F3 could promote ECs angiogenesis *in vivo* [49] and glioma cells could also enhance ECs angiogenesis by activating VEGFA and TGFβ through the release of exosomes containing lncRNA CCAT2 [55]. Moreover, Kaneko et al demonstrated that Meg3 epigenetically regulated gene expression via interacting with Ezh2 and Jarid2 [56], and Boon et al. further reported that Meg3-mediated changes in epigenetic regulation of gene expression contributed to endothelial dysfunction in angiogenesis [57].

Except from the role in regulating angiogenesis of ECs, CCAT2 could also inhibit ECs apoptosis by decreasing Bax and caspase-3 expression [55]. Conversely, recent report had shown that high expression of lncRNA GAS5 could increase the apoptosis of vascular ECs [58]. Meanwhile, MALAT1, involved in modulating ECs proliferation as mentioned before [50], had been also identified as a regulator of inflammatory cytokine production and affected inflammation of ECs through SAA3 [59, 60].

### 2.2 Exosomal ncRNAs and VSMCs functions

VSMCs, the main cells of the media vessel wall, can control blood flow and keep vascular tension maintenance. VSMCs phenotypic conversion from a contractile to synthetic state with the progress of aging and in response to various pathological stimuli, contributes to vascular pathologies including atherosclerosis, restenosis, and VC. Investigators indicated that exosomes acted as a specific signaling mechanism during VSMC phenotypic modulation was taken part in regulating cellular proliferation, migration, apoptosis, calcification, and differentiation [61]. In this part, we summarized the role of exosomal ncRNAs in the regulation of VSMCs functions (Table 2).

#### 2.2.1 Exosomal miRNAs and VSMCs functions

Previous studies had indicated that several exosomal miRNAs were involved in the regulation of VSMCs phenotypic modulation. The contractile phenotype of VSMCs with non-proliferative and non-migratory switched to a proliferative, migratory, apoptotic, and differentiation phenotype [61, 62].

Liu et al. had proved that exosomal miR-31 could promote the VSMCs contractile phenotype by repressing cellular repressor of E1A-stimulated genes (CREG) expression [63]. Besides, exosomes containing miR-133 had been shown to regulate the differentiation of VSMCs by inhibiting the transcription factor, specificity protein-1 (Sp-1) [64]. In addition, it had been reported that miR-223 regulated VSMCs phenotype transition from contractile to synthesis [65]. On the contrary, miR-26a inhibited the differentiation of VSMCs and governed phenotypic shifting by directly targeting SMAD1 and SMAD4 [66].

An increasing number of studies had demonstrated that miRNAs played a pivotal role in the progress of VSMCs proliferation through post-transcriptional mechanisms [67, 68]. For example, some exosomal miRNAs had been found to promote the proliferation of VSMCs such as miR-21, miR-130a, miR-221/222, et al [67-69]. MiR-21 is one of the most abundant miRNAs in the vascular wall which can enhance the VSMCs proliferation [67]. miR-221 and miR-222 had been shown to regulate the proliferation of VSMCs by targeting p27 and p57 [69, 70]. While, others had been proven to inhibit the proliferation of VSMCs [71, 72]. MiR-152 inhibited cell proliferation in VSMCs by decreasing DNA methyltransferase 1 (DNMT1) expression and downregulating the methylation level of ERα gene promoter region [73]. MiR-155 induced by oxidized low-density lipoprotein (ox-LDL) was considered to inhibit the proliferation of VSMCs by suppression of certain members of the matrix metalloproteinase (MMP) family [74]. Besides, Tan et al. indicated that activated platelet-derived exosomes containing miR-223, miR-339 and miR-21 could be transferred into VSMCs and inhibit VSMCs proliferation [75].

**Table 2:** Exosomal non-coding RNAs implicated in VSMCs functions.

Exosomal non-coding RNAs	Cargos	Targets	ECs functions	Reference
Exosomal miRNAs	miR-31	MAPK/ERK, LATS2	promote phenotype transition	[63]
	miR-133	Sp1	promote phenotype transition	[64]
	miR-223	MEF2C, RhoB	promote phenotype transition	[65]
	miR-26a	Smad1	inhibit phenotype transition	[66]
	miR-21	BCL2	pro-proliferation	[67]
	miR-130a	BMP2, TGFβ1	pro-proliferation	[68]
	miR-221/222	p27, p57	pro-proliferation	[69, 70]
	miR-152	DNMT1	anti-proliferation	[73]
	miR-155	Ets1	anti-proliferation	[74]
	miR-223	PDGFRβ	anti-proliferation	[75]
	miR-339	PDGFRβ	anti-proliferation	[75]
	miR-21	PDGFRβ	anti-proliferation	[75]
	miR-29b	DNMT3b	pro-migration	[78]
	miR-143-3p	TGFβ	pro-migration, pro-angiogenesis	[79]
	miR-712	NCKX4	pro-calcification	[80]
	miR-714	PMCA1	pro-calcification	[80]
	miR-762	NCX1	pro-calcification	[80]
	miR-29a/b	ADAMTS-7	anti-calcification	[83]
	miR-125b	Ets1, Osterix	anti-transdifferentiation, anti-calcification	[84, 85]
	miR-126-3p	VEGF, ANG1, ANG2, MMP9, TSP1	pro-angiogenesis	[86]
	miR-92a	MKK4	anti-apoptosis, anti-senescence	[88]
	miR-34a	SIRT1	pro-senescence	[89]
Exosomal lncRNAs	MALAT1	Unknown	pro-proliferation, pro-migration	[92]
	MEG3	p53	anti-proliferation, anti-migration	[93]
	lncRNA-p21	p53	anti-proliferation, pro-apoptosis	[94]
	HOTAIR	*ALPL, BMP2*	anti-calcification	[95]

MAPK/ERK: mitogen-activated protein kinase/extracellular regulated kinase; LATS2: large tumor suppressor homolog 2; Sp1: specificity protein 1; MEF2C: myocyte enhancer factor-2c; RhoB: ras homologue family member B; Smad1: SMAD family member 1; BCL2: B-cell CLL/lymphoma 2; BMP2: bone morphogenetic protein 2; TGFβ1: transforming growth factor beta 1; DNMT1: DNA methyltransferase 1; Ets1: E26 transformation-specific 1; PDGFRβ: platelet-derived growth factor receptor-beta; DNMT3b: DNA methyltransferase 3b; TGFβ:transforming growth factor β; NCKX4: sodium/calcium exchanger 4; PMCA1: plasma membrane calcium ATPase 1; NCX1: sodium/calcium exchanger 1; ADAMTS-7: a disintegrin and metalloproteinase with thrombospondin motifs-7; VEGF: vascular endothelial growth factor; ANG1: angiopoietin 1; ANG2: angiopoietin 2; MMP9: matrix metallopeptidase 9; TSP1: thrombospondin 1; MKK4: mitogen-activated protein kinase 4; SIRT1: silent information regulator 1; ALPL: alkaline phosphatase; BMP2: bone morphogenetic Protein 2.

VSMCs can migrate from the medial wall to the intimal lining in response to vascular injury. Some miRNAs involved in the regulatory mechanisms of cellular migration are promoters while others are inhibitors [76, 77]. For instance, Chen et al. indicated that miR-29b promoted VSMCs migration by regulating the expression of MMP-2/MMP-9 genes and targeting DNA methyltransferase 3b (DNMT3b) [78]. In addition, miR-143-3p could modulate exosome-mediated responses in pulmonary VSMCs and Deng at al. proved that miR-143-3p enriched exosomes had the VSMCs promigratory and proangiogenic effect [79].

VC is a tightly regulated process actually driven by VSMCs osteogenic conversion and related to the activation of sphingomyelin phosphodiesterase 3 (SMPD3) and cytoskeletal remodeling. As the process of VSMCs calcification is tightly regulated by the genetic reprogramming, it is not surprising that there are accumulating evidence to support an integral role of miRNAs involved in this process [80-82]. One study reported that several exosomal miRNAs could disrupt calcium transporters and promote calcium deposition of VSMCs including miR-712, miR-714 and miR-762 [80]. On the contrary, Du et al. identified that miR-29a/b inhibited VSMCs calcification by suppressing the expression of a disintegrin and metalloproteinase with thrombospondin motifs-7 (ADAMTS-7) [83]. Moreover, the expression of miR-125b suppressed VSMCs transdifferentiation and calcification [84, 85].

Angiogenesis of VSMCs is responsible for the progression of vascular aging. MiR-126-3p enhanced VSMCs angiogenesis by suppressing the expression of its known target, SPRED1, simultaneously modulating the expression of genes involved in angiogenic pathways including vascular endothelial growth factor (VEGF), angiopoietin 1 (ANG1), angiopoietin 2 (ANG2), matrix metallopeptidase 9 (MMP9), thrombospondin 1 (TSP1) and so on [86]. Apoptosis and senescence of VSMCs had been proven as important processes in vascular diseases [87]. It had been shown that miR-92a overexpression inhibited VSMCs apoptosis and senescence by suppressing mitogen-activated protein kinase 4 (MKK4) and JNK1 pathways [88]. In contrast, miR-34a could promote VSMCs senescence through SIRT1 downregulation [89].

#### 2.2.2 Exosomal lncRNAs and VSMCs functions

LncRNAs are capable of modulating target DNA, RNA, and proteins at the pre- and posttranscriptional level. An increasing number of studies had demonstrated that lncRNAs played a pivotal role in the regulation of VSMC phenotype, functions, perhaps also in the development of VSMCs-related diseases [90, 91]. This chapter described the emerging roles of exosomal lncRNAs in VSMCs proliferation, migration, apoptosis, and calcification.

As mentioned above, MALAT1 could regulate the proliferative and promigratory functions of ECs [50], Brock et al. [92] proved that MALAT1 could also significantly enhance proliferation and migration of pulmonary VSMCs, probably through hypoxia-inducible factor 1α. In addition, Sun et al. [93] found that inhibition of MEG3 regulated the cell cycle progression and promoted more VSMCs from the G0/G1 phase to the G2/M+S phase. Therefore, lncRNA MEG3 downregulation triggers pulmonary VSMCs proliferation and migration. Exosomal lncRNA-p21 was a p53-induced lncRNA [45], which recently was proven to control VSMCs proliferation [94]. Importantly, subsequent studies showed that inhibition of lncRNA-p21 could also reduce apoptosis of VSMCs by interfering with p53 [94]. Besides, Carrion et al. showed that silencing of lncRNA HOTAIR elevated expressions of calcification-related genes, such as BMP2 and ALP, which eventually played a key role in calcification of VSMCs [95].

**Figure 1.**
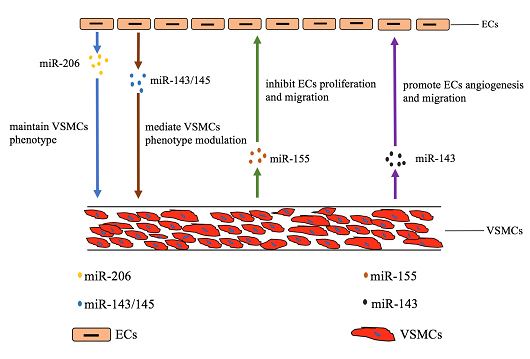
**The communication between ECs and VSMCs via exosomal ncRNAs**. The blue and brown arrows indicate that exosomal miR206 and miR-143/145 secreted by ECs regulate the functions of VSMCs. The green and purple arrows indicate that exosomal miR-155 and miR-143 secreted by VSMCs regulate the functions of ECs.

## 3. The role of exosomes in the network of information communication between ECs and VSMCs

Exosomes, a newly identified natural nanocarrier and intercellular messenger, played a pivotal role in regulating cell-to-cell communication. Active interactions between ECs and VSMCs are critical to modulate the process of vascular aging. Accumulating evidence showed that the close communication between ECs and VSMCs were achieved via the release of exosomes [96, 97]. However, little is known about the mechanisms that regulate ECs and VSMCs crosstalk. In this part, we aimed to discuss the mechanisms underlying the regulation of the ECs and VSMCs through exosomes especially exosomal ncRNAs (Figure 1).

Several reports showed that ECs could regulate the functions of VSMCs through the release of exosomes containing miRNAs. For example, the expression of the miR-143/145 cluster was regulated by shear-responsive transcription factor KLF2 in ECs [97] and then miR-143/145 could mediate VSMCs phenotype modulation by transporting to VSMCs via exosomes [98, 99]. In addition, miR-206 derived from HUVEC maintains the contractile phenotype of VSMCs by suppressing exosomes secretion [100].

A large line of reports found that VSMCs could also modulate ECs functions through exosomes. For instance, miR-155, significantly expressed in Krüppel-like factor 5 (KLF5)-overexpressing VSMCs, was a potent regulator of endothelium barrier function. VSMCs-derived exosomes mediated the transfer of miR-155 from VSMCs to ECs, which, in turn, inhibited EC proliferation and migration, thus eventually destroying the tight junctions and the integrity of endothelial barriers [101]. Deng et al. had discovered that a crucial exosomal communication between pulmonary VSMCs and ECs in pulmonary artery hypertension (PAH) [79]. It had been proven that exosomes secreted by pulmonary VSMCs were enriched with miR-143, which could be transported to ECs, inducing ECs migration and angiogenesis [79].

**Table 3:** Roles of exosomal miRNAs in vascular aging related diseases.

	Cargo	Disease	Functions	
Heart Diseases	miR-221/222	CAD	Promote the progression of CAD	[103]
	miR-208a	CAD	Promote the progression of CAD	[104]
	miR-126	CAD	Inhibit the progression of CAD	[105]
	miR-17-92	CAD	Inhibit the progression of CAD	[106]
	miR-22	AMI	Protect against CMCs apoptosis	[108]
	miR-133	AMI	Promote the progression of AMI	[109]
	miR-21	AMI, HF	Promote CMCs loss	[110, 113]
	miR-24	HF	Promote the progression of HF	[114]
	miR-214	HF	Promote the progression of HF	[115]
Hypertension	miR-211	EH	Activate the axis of RAAS	[119]
	miR-615	EH	Activate the axis of RAAS	[119]
	miR-155	EH	Regulate the progression of VC	[99]
Cerebrovascular Diseases	miR-126	AIS	Inhibit microglial activation and inflammatory response	[122]
	miR-30d-5p	AIS	Inhibit autophagy-mediated microglial polarization to M1	[123]
	miR-181b-5p	AIS	promoted BMEC angiogenesis	[37]
	miR-146a	VCID	Inhibit inflammatory effects on damaged astrocytes	[124]
Kidney Diseases	miR-200b	CKD	Regulate the progression of renal fibrosis	[126]
	miR-16	CKD	Regulate the progression of CKD	[127]
PAD	miR-92a	Hind Limb Ischemia	Inhibit functional recovery	[129]

CAD: coronary artery disease; AMI: acute myocardial infarction; CMCs: cardiomyocytes; HF: heart failure; VC: vascular calcification; EH: essential hypertension; RAAS: rennin-angiotensin-aldosterone system; AIS: acute ischemic stroke; BMEC: brain microvascular endothelial cell; VCID: vascular cognitive impairment and dementia; CKD: chronic kidney disease; PAD: peripheral artery disease.

## 4.The role of exosomes in vascular aging related diseases

Vascular aging occurs earlier than clinical diseases. It is a high-risk factor of vascular aging-related diseases. Vascular aging and vascular diseases interact with each other. The aging blood vessels provide an environment for the occurrence and development of vascular diseases, whereas vascular diseases accelerate the process of vascular aging. Exosomal non-coding RNAs are recently regarded as a critical factor in vascular diseases. It has been demonstrated that a number of vascular aging related diseases like heart diseases, hypertension, cerebro-vascular diseases, kidney diseases, and peripheral artery diseases (PAD) are highly influenced by exosomes (Table 3).

### 4.1 The role of exosomes in vascular aging related heart diseases

Intercellular communication mediated by exosomes is vital for preserving vascular integrity and in the development of cardiovascular diseases. A recent surge of reports suggested that major heart diseases like coronary artery disease (CAD), acute myocardial infarction (AMI) and heart failure (HF) were highly influenced by the cargoes in exosomes.

The present study demonstrated that vessel wall and inflammatory cell-derived miRNAs could be detected in the blood and were regulated in patients with CAD. The communication among the involved cells, leading to promote the formation of plaque, were regulated by exosomes in CAD [102]. Bazan et al. found that exosomes derived from VSMCs exhibited more pro-atherosclerotic miR-221/222 in patients with CAD [103]. MiR-208a,which was enriched in serum-derived exosomes,tended to be elevated with stable CAD [104]. Vascular miRNAs, including miR-126 [105] and miR-17-92 cluster [106], were downregulated in patients with CAD.

AMI, a detrimental consequence of acute coronary occlusion, is characterized by cardiomyocyte loss and myocardial necrosis [107]. It had been revealed that exosomes contained the increased amount of miR-22 under ischemic condition, and could be centralized to cardiomyocytes to protect against apoptosis by targeting methyl-CpG-binding protein 2 [108]. On the contrary, it had been proved that exosomal miR-133 [109] and miR-21 [110] could promote the progression of AMI.

HF is a general term for the heart’s inability to pump sufficiently and maintain blood flow to meet the body’s needs. In general, significant cardiac ultrastructural, biochemical and molecular abnormalities, contribute to the pathophysiology of all forms of HF [111]. A number of exosomal miRNAs were recurrent [112] and confirmed to be altered in various forms of HF. It had been reported that miR-21 [113], miR-24 [114] and miR-214 [115] were consistently affected in patients with varying origins and degrees of HF.

### 4.2 The role of exosomes in vascular aging related primary hypertension

Primary hypertension is characterized by decreased compliance and increased rigidity of vessels. The interaction between vascular aging and hypertension contributes to arterial stiffness, while arterial stiffness and hypertension are mutually causal. On the one hand, hypertension causes vessel wall injury, leading to arterial stiffness. On the other hand, arterial stiffness is the main cause of high systolic blood pressure, especially in the elderly [116]. Activation of rennin-angiotensin-aldosterone system (RAAS) is the bedrock in hypertension [117]. Recently, it had been demonstrated that exosomal miRNAs were altered by RAAS activation and thus correlated with hypertension [118]. The miRNome analysis of exosomes from patients with hypertension found that miR-211 and miR-615 were fluctuated with blood pressure [119]. Besides, VC, the hydroxyapatite deposition in the intimal and medial layers of the vascular wall, can lead to hypertension. Chen et al. reported that miR-155 could regulate VC procession [99].

### 4.3 The role of exosomes in vascular aging related cerebrovascular diseases

Vascular aging leads to the development of hemodynamic aging syndrome [120]. It may cause cerebrovascular diseases and cognitive impairment because of the high pulsation blood flow.

Acute ischemic stroke and lacunar infarction are associated with endothelial dysfunction. Vascular aging may play an important role in the pathogenesis of stroke and serve as a potential marker of the risk of stroke [121]. Geng et al. found that miR-126 was significantly reduced in patients with ischemic stroke, miR-126^+^ exosomes could inhibit microglial activation and inflammatory response induced by ischemic stroke and promote neurogenesis and vasculogenesis after ischemic stroke [122]. Jiang et al. revealed that exosomes enriched with miR-30d-5p had a protective effect on AIS by inhibiting autophagy-mediated microglial polarization to M1 [123]. Yang et al. suggested that the expression of miR-181b-5p were remarkably enhanced in exosomes, which indicated that miR-181b-5p may involve in the progress of AIS [37].

Vascular cognitive impairment and dementia is a type of cognitive disorder induced by vascular abnormalities. It had been reported that exosomal miR-146a exerted anti-inflammatory effects on damaged astrocytes and prevented diabetes-induced cognitive impairment [124].

### 4.4 The role of exosomes in vascular aging related kidney diseases

The interaction of vascular aging and arteriosclerosis can ultimately contribute to chronic kidney disease (CKD) [125]. Arteriosclerosis is common in end-stage renal disease (ESRD). The incidence of plaque calcification and arterial stiffness is increased in patients with ESRD. VC is a strong predictor and common complication in patients with ESRD. Yu et al. found that the renal tubule-derived urinary exosomal miR-200b could regulate the progression of renal fibrosis, eventually leading to CKD [126]. Lange et al. concluded that miR-16 derived from urinary exosomes was the most stable endogenous reference gene in patients with CKD [127].

### 4.5 The role of exosomes in vascular aging related PAD

PAD is a vascular abnormity of diffuse atherosclerosis and is a major cause of cardiovascular mortality [128]. To date, less attention were paid to the association of miRNAs with vascular integrity in PAD. Bonauer et al. revealed that the expression of exosomal miR-92a was significantly increased in ischemic injury. Besides, inhibition of miR-92a promoted functional recovery in mice with ischemic damage to limb muscle [129].

## 5.Diagnosis and clinical evaluation of vascular aging

The specific cargoes of exosomes make them to be promising novel biomarkers and diagnostic tool for vascular aging. It especially concerns exosomes-associated miRNAs because of the significantly different levels between physiological and pathological conditions. In patients affected with AMI, the level of p53 responsive miRs (miR-192, miR-194, miR-34a) inside in exosomes were associated with development of HF, which suggesting a diagnostic value of these miRNAs [130]. The miRNome analysis of exosomes from hypertensive patients proved that several miRNAs including miR-615, miR-211 were sensitive to blood pressure, which exhibited a promising prognostic biomarker on hypertension [119]. Nevertheless, evaluation of exosomes as a source of diagnostic and prognostic biomarkers of vascular diseases is still in its infancy. Further studies should be done to validate diagnostic and prognostic value of exosome-associated biomarkers.

Clinical evaluation of vascular aging is generally divided into invasive and non-invasive methods. Invasive evaluations are rarely used in clinical practice because of their complex requirements, high cost and damage. Currently, non-invasive methods are commonly used in clinical assessment of vascular aging mainly include: (1) Framingham formula; (2) non-invasive detection of vascular function and structure; (3) biomarkers of vascular senescence cells [131].

(1) *Framingham formula:* Framingham heart study establishes a vascular age formula based on risk factors for cardiovascular events, such as men, hyperlipidemia, hypertension, diabetes, and smoking. It proposes the concept of vascular age, which is still widely used in clinical vascular age assessment and guidance of cardiovascular risk prevention [132].
(2) *Non-invasive detection of vascular function and structure:* (i) Pulse wave velocity (PWV) is the conduction velocity of the arterial pulse wave along the arterial wall from the proximal to the distal end when the heart pump hemorrhage occurs. The ratio of the distance between the two recorded parts and the conduction time of the pulse wave is measured. PWV is based on the mechanism that the speed of conduction of pulse waves in the blood output from the heart increases when the arteriosclerosis is hardened. The more severe arterial stiffness becomes, the greater the PWV is. The elastic components of the aorta decrease, the vessel wall thickens, the arteries expand progressively, the arterial stiffness increases, and the PWV increases with increasing aging. Moreover, the PWV of central elastic artery increased more significantly than that of peripheral artery [133]. PWV is positively correlated with Framingham and has predictive value for the diagnosis of coronary heart disease [134]. (ii) Intima-media thickness (IMT) is the distance between the arterial intima to media that measured by high-frequency B-mode ultrasound probe. IMT thickening is the landmark of vascular aging. The carotid IMT is an independent predictor of cardiovascular and cerebrovascular risk. For each 0.1 mm increase in carotid IMT, the risk of myocardial infarction increased by 11% [135]. IMT in healthy people is increasing gradually with the increase of age. Besides, vascular aging is accelerated as well as IMT is growing faster in the populations with atherosclerotic cardiovascular risk factors. (iii) The principle of vasodilation function is to measure the temporal change of brachial artery diameter under shear stress by ultrasound. The impaired diastolic function of brachial artery was associated with aging [136].
(3) *Biomarkers of vascular senescence cells:* Currently, endothelial microparticles (EMPs) and endothelial progenitor cells (EPCs) are widely recognized and detected as cell markers. EMPs are microparticles carrying certain antigenic properties of ECs when they are activated, damaged or apoptotic. The increase of EMPs represents vascular endothelial injury, indicating vascular aging [137]. When vascular endothelial injury occurs, EPCs, derived from bone marrow, could be differentiated into mature ECs to repair damage blood vessels. Besides, EPCs can activate the proliferation and migration of ECs around the injured region through paracrine secretion and it is an important mechanism of endogenous vascular endothelial repair [138]. The decreasing of EPCs is closely related to the prognosis of atherosclerosis and cardiovascular diseases, which suggesting vascular aging [139].

## 6.Treatment and clinic intervention of vascular aging

In addition to act as potential biomarkers, exosomes could also be served as a therapeutic tool for treatment of vascular diseases. The main promise of using exosomes as a therapeutic tool is their property to stably maintain and carry a variety of biomolecules. Specific gene expression can be modulated by genetic approaches including silencing or overexpression of the prospective miRNAs or lncRNAs. The promising miRNA triad (miR-126, miR-145 and miR-155) could be primarily suggested for anti-atherogenic therapy. Moreover, since exosomes possess some properties such as biological barrier permeability, biocompatibility, low immunogenicity and low toxicity, which are suitable for therapeutic delivery [140, 141]. Thus, many researches investigate the function of exosomes as vehicles for exogenous miRNAs, lncRNAs, and even drugs delivery. Notably, although targeting miRNAs or lncRNAs shows promising therapeutic strategies, careful monitoring and studying of these interactions is essential and important in order to guarantee a safe application in humans.

Vascular aging is not only a natural physiological process, but also a pathological process involving a variety of risk factors, among which genetics, environment and lifestyle are involved in. It is an effective means of anti-vascular aging in clinic that regulating the reversible mechanism of vascular aging and strengthening the prevention and control of risk factors. The patients with vascular aging had better improve the lifestyle through healthy eating, weight control, smoking cessation and reasonable exercise. Drugs target for risk factors such as angiotensin-converting enzyme inhibitors, calcium antagonists or endothelial function like beraprost sodium have been found to improve vascular function, ameliorate arterial stiffness, and regulate the process of vascular aging. Although aging is irreversible, early detection and intervention of premature vascular failure is a new direction for prevention and treatment of cardiovascular, cerebrovascular and other vascular diseases.

## 7.Perspectives and Conclusions

The accumulation of the changes in the structure and function of aging vessels constitute the basis of vascular aging, while vascular aging is the basis of a variety of vascular diseases. Therefore, better understanding of the mechanism, evaluation and management of vascular aging will provide new research targets for vascular diseases. The cargos of exosomes especially ncRNAs play a crucial role in regulating aging processes and vascular aging related diseases. It also offers promising opportunities for treating and assessing vascular diseases. Despite interesting promises in implication, exosomal ncRNAs-based therapies still require more preclinical studies and many obstacles still need to be overcome in the future.

## References

[1] Wu M, Rementer C, Giachelli CM (2013). Vascular calcification: an update on mechanisms and challenges in treatment. Calcif Tissue Int, 93: 365-73. 23456027 10.1007/s00223-013-9712-z PMC3714357

[2] Lin X, Zhan JK, Wang YJ, Tan P, Chen YY, Deng HQ, et al (2016). Function, Role, and Clinical Application of MicroRNAs in Vascular Aging. Biomed Res Int, 2016: 6021394. 28097140 10.1155/2016/6021394 PMC5209603

[3] Jenjaroenpun P, Kremenska Y, Nair VM, Kremenskoy M, Joseph B, Kurochkin IV (2013). Characterization of RNA in exosomes secreted by human breast cancer cell lines using next-generation sequencing. PeerJ, 1: e201. 24255815 10.7717/peerj.201 PMC3828613

[4] Lai RC, Chen TS, Lim SK (2011). Mesenchymal stem cell exosome: a novel stem cell-based therapy for cardiovascular disease. Regen Med, 6: 481-92. 21749206 10.2217/rme.11.35

[5] Liao XB, Zhang ZY, Yuan K, Liu Y, Feng X, Cui RR, et al (2013). MiR-133a modulates osteogenic differentiation of vascular smooth muscle cells. Endocrinology, 154: 3344-52. 23798596 10.1210/en.2012-2236

[6] Cui RR, Li SJ, Liu LJ, Yi L, Liang QH, Zhu X, et al (2012). MicroRNA-204 regulates vascular smooth muscle cell calcification in vitro and in vivo. Cardiovasc Res, 96: 320-9. 22871591 10.1093/cvr/cvs258

[7] Liu FJ, Wen T, Liu L (2012). MicroRNAs as a novel cellular senescence regulator. Ageing Res Rev, 11: 41-50. 21689787 10.1016/j.arr.2011.06.001

[8] Dimmeler S, Nicotera P (2013). MicroRNAs in age-related diseases. EMBO Mol Med, 5: 180-90. 23339066 10.1002/emmm.201201986 PMC3569636

[9] Kapustin AN, Shanahan CM (2016). Emerging roles for vascular smooth muscle cell exosomes in calcification and coagulation. J Physiol, 594: 2905-14. 26864864 10.1113/JP271340 PMC4887700

[10] Zhang C, Zhang K, Huang F, Feng W, Chen J, Zhang H, et al (2018). Exosomes, the message transporters in vascular calcification. J Cell Mol Med, 22: 4024-4033. 29892998 10.1111/jcmm.13692 PMC6111818

[11] Yan B, Wang Z (2012). Long noncoding RNA: its physiological and pathological roles. DNA Cell Biol, 31 Suppl 1: S34-41. 22612272 10.1089/dna.2011.1544

[12] Gupta SK, Piccoli MT, Thum T (2014). Non-coding RNAs in cardiovascular ageing. Ageing Res Rev, 17: 79-85. 24491506 10.1016/j.arr.2014.01.002

[13] Maiese K (2017). Harnessing the Power of SIRT1 and Non-coding RNAs in Vascular Disease. Curr Neurovasc Res, 14: 82-88. 27897112 10.2174/1567202613666161129112822 PMC5383524

[14] He C, Zheng S, Luo Y, Wang B (2018). Exosome Theranostics: Biology and Translational Medicine. Theranostics, 8: 237-255. 29290805 10.7150/thno.21945 PMC5743472

[15] O'Loughlin AJ, Woffindale CA, Wood MJ (2012). Exosomes and the emerging field of exosome-based gene therapy. Curr Gene Ther, 12: 262-74. 22856601 10.2174/156652312802083594

[16] Tabas I, Garcia-Cardena G, Owens GK (2015). Recent insights into the cellular biology of atherosclerosis. J Cell Biol, 209: 13-22. 25869663 10.1083/jcb.201412052 PMC4395483

[17] Gimbrone MA Jr, Garcia-Cardena G (2016). Endothelial Cell Dysfunction and the Pathobiology of Atherosclerosis. Circ Res, 118: 620-36. 26892962 10.1161/CIRCRESAHA.115.306301 PMC4762052

[18] Staszel T, Zapala B, Polus A, Sadakierska-Chudy A, Kiec-Wilk B, Stepien E, et al (2011). Role of microRNAs in endothelial cell pathophysiology. Pol Arch Med Wewn, 121: 361-6. 21946298

[19] Das S, Halushka MK (2015). Extracellular vesicle microRNA transfer in cardiovascular disease. Cardiovasc Pathol, 24: 199-206. 25958013 10.1016/j.carpath.2015.04.007

[20] Kishore R, Garikipati VN, Gumpert A (2016). Tiny Shuttles for Information Transfer: Exosomes in Cardiac Health and Disease. J Cardiovasc Transl Res, 9: 169-75. 26911150 10.1007/s12265-016-9682-4 PMC4874852

[21] Santulli G (2016). MicroRNAs and Endothelial (Dys) Function. J Cell Physiol, 231: 1638-44. 26627535 10.1002/jcp.25276 PMC4871250

[22] Araldi E, Suarez Y (2016). MicroRNAs as regulators of endothelial cell functions in cardiometabolic diseases. Biochim Biophys Acta, 1861: 2094-2103. 26825686 10.1016/j.bbalip.2016.01.013 PMC5039046

[23] Jia L, Zhou X, Huang X, Xu X, Jia Y, Wu Y, et al (2018). Maternal and umbilical cord serum-derived exosomes enhance endothelial cell proliferation and migration. Faseb j, 32: 4534-4543. 29570394 10.1096/fj.201701337RR

[24] van Balkom BW, de Jong OG, Smits M, Brummelman J, den Ouden K, de Bree PM, et al (2013). Endothelial cells require miR-214 to secrete exosomes that suppress senescence and induce angiogenesis in human and mouse endothelial cells. Blood, 121: 3997-4006, s1-15. 23532734 10.1182/blood-2013-02-478925

[25] Garcia NA, Ontoria-Oviedo I, Gonzalez-King H, Diez-Juan A, Sepulveda P (2015). Glucose Starvation in Cardiomyocytes Enhances Exosome Secretion and Promotes Angiogenesis in Endothelial Cells. PLoS One, 10: e0138849. 26393803 10.1371/journal.pone.0138849 PMC4578916

[26] Iaconetti C, Polimeni A, Sorrentino S, Sabatino J, Pironti G, Esposito G, et al (2012). Inhibition of miR-92a increases endothelial proliferation and migration in vitro as well as reduces neointimal proliferation in vivo after vascular injury. Basic Res Cardiol, 107: 296. 22890560 10.1007/s00395-012-0296-y

[27] Jin C, Zhao Y, Yu L, Xu S, Fu G (2013). MicroRNA-21 mediates the rapamycin-induced suppression of endothelial proliferation and migration. FEBS Lett, 587: 378-85. 23313253 10.1016/j.febslet.2012.12.021

[28] Svensson D, Gidlof O, Turczynska KM, Erlinge D, Albinsson S, Nilsson BO (2014). Inhibition of microRNA-125a promotes human endothelial cell proliferation and viability through an antiapoptotic mechanism. J Vasc Res, 51: 239-45. 25116893 10.1159/000365551

[29] Qin G, Dong Z, Zeng P, Liu M, Liao X (2013). Association of vitamin D receptor BsmI gene polymorphism with risk of osteoporosis: a meta-analysis of 41 studies. Mol Biol Rep, 40: 497-506. 23054016 10.1007/s11033-012-2086-x

[30] Loyer X, Potteaux S, Vion AC, Guerin CL, Boulkroun S, Rautou PE, et al (2014). Inhibition of microRNA-92a prevents endothelial dysfunction and atherosclerosis in mice. Circ Res, 114: 434-43. 24255059 10.1161/CIRCRESAHA.114.302213

[31] Zhou J, Wang KC, Wu W, Subramaniam S, Shyy JY, Chiu JJ, et al (2011). MicroRNA-21 targets peroxisome proliferators-activated receptor-alpha in an autoregulatory loop to modulate flow-induced endothelial inflammation. Proc Natl Acad Sci U S A, 108: 10355-60. 21636785 10.1073/pnas.1107052108 PMC3121870

[32] Goodwin AJ, Guo C, Cook JA, Wolf B, Halushka PV, Fan H (2015). Plasma levels of microRNA are altered with the development of shock in human sepsis: an observational study. Crit Care, 19: 440. 26683209 10.1186/s13054-015-1162-8 PMC4699334

[33] Real JM, Ferreira LRP, Esteves GH, Koyama FC, Dias MVS, Bezerra-Neto JE, et al (2018). Exosomes from patients with septic shock convey miRNAs related to inflammation and cell cycle regulation: new signaling pathways in sepsis? Crit Care, 22: 68. 29540208 10.1186/s13054-018-2003-3 PMC5852953

[34] Li J, Tan M, Xiang Q, Zhou Z, Yan H (2017). Thrombin-activated platelet-derived exosomes regulate endothelial cell expression of ICAM-1 via microRNA-223 during the thrombosis-inflammation response. Thromb Res, 154: 96-105. 28460288 10.1016/j.thromres.2017.04.016

[35] Lee C, Mitsialis SA, Aslam M, Vitali SH, Vergadi E, Konstantinou G, et al (2012). Exosomes mediate the cytoprotective action of mesenchymal stromal cells on hypoxia-induced pulmonary hypertension. Circulation, 126: 2601-11. 23114789 10.1161/CIRCULATIONAHA.112.114173 PMC3979353

[36] Liang X, Zhang L, Wang S, Han Q, Zhao RC (2016). Exosomes secreted by mesenchymal stem cells promote endothelial cell angiogenesis by transferring miR-125a. J Cell Sci, 129: 2182-9. 27252357 10.1242/jcs.170373

[37] Yang Y, Cai Y, Zhang Y, Liu J, Xu Z (2018). Exosomes Secreted by Adipose-Derived Stem Cells Contribute to Angiogenesis of Brain Microvascular Endothelial Cells Following Oxygen-Glucose Deprivation In Vitro Through MicroRNA-181b/TRPM7 Axis. J Mol Neurosci, 65: 74-83. 29705934 10.1007/s12031-018-1071-9

[38] Li Y, Liang J, Hu J, Ren X, Sheng Y (2018). Down-regulation of exosomal miR-106b-5p derived from cholesteatoma perimatrix fibroblasts promotes angiogenesis in endothelial cells by overexpression of Angiopoietin 2. Cell Biol Int, 42: 1300-1310. 29905392 10.1002/cbin.11002

[39] Wang X, Huang W, Liu G, Cai W, Millard RW, Wang Y, et al (2014). Cardiomyocytes mediate anti-angiogenesis in type 2 diabetic rats through the exosomal transfer of miR-320 into endothelial cells. J Mol Cell Cardiol, 74: 139-50. 24825548 10.1016/j.yjmcc.2014.05.001 PMC4120246

[40] Chen Y, Li D, Xu Y, Zhang Y, Tao L, Li S, et al (2014). Essential Oils from Fructus A. zerumbet Protect Human Aortic Endothelial Cells from Apoptosis Induced by Ox-LDL In Vitro. Evid Based Complement Alternat Med, 2014: 956824. 25610487 10.1155/2014/956824 PMC4290151

[41] Hung T, Chang HY (2010). Long noncoding RNA in genome regulation: prospects and mechanisms. RNA Biol, 7: 582-5. 20930520 10.4161/rna.7.5.13216 PMC3073254

[42] Rinn JL, Chang HY (2012). Genome regulation by long noncoding RNAs. Annu Rev Biochem, 81: 145-66. 22663078 10.1146/annurev-biochem-051410-092902 PMC3858397

[43] Wang KC, Chang HY (2011). Molecular mechanisms of long noncoding RNAs. Mol Cell, 43: 904-14. 21925379 10.1016/j.molcel.2011.08.018 PMC3199020

[44] Khaitan D, Dinger ME, Mazar J, Crawford J, Smith MA, Mattick JS, et al (2011). The melanoma-upregulated long noncoding RNA SPRY4-IT1 modulates apoptosis and invasion. Cancer Res, 71: 3852-62. 21558391 10.1158/0008-5472.CAN-10-4460

[45] Huarte M, Guttman M, Feldser D, Garber M, Koziol MJ, Kenzelmann-Broz D, et al (2010). A large intergenic noncoding RNA induced by p53 mediates global gene repression in the p53 response. Cell, 142: 409-19. 20673990 10.1016/j.cell.2010.06.040 PMC2956184

[46] Tian D, Sun S, Lee JT (2010). The long noncoding RNA, Jpx, is a molecular switch for X chromosome inactivation. Cell, 143: 390-403. 21029862 10.1016/j.cell.2010.09.049 PMC2994261

[47] Yin DD, Zhang EB, You LH, Wang N, Wang LT, Jin FY, et al (2015). Downregulation of lncRNA TUG1 affects apoptosis and insulin secretion in mouse pancreatic beta cells. Cell Physiol Biochem, 35: 1892-904. 25871529 10.1159/000373999

[48] Liao B, Chen R, Lin F, Mai A, Chen J, Li H, et al (2018). Long noncoding RNA HOTTIP promotes endothelial cell proliferation and migration via activation of the Wnt/beta-catenin pathway. J Cell Biochem, 119: 2797-2805. 29058802 10.1002/jcb.26448

[49] Lang HL, Hu GW, Chen Y, Liu Y, Tu W, Lu YM, et al (2017). Glioma cells promote angiogenesis through the release of exosomes containing long non-coding RNA POU3F3. Eur Rev Med Pharmacol Sci, 21: 959-972. 28338200

[50] Michalik KM, You X, Manavski Y, Doddaballapur A, Zornig M, Braun T, et al (2014). Long noncoding RNA MALAT1 regulates endothelial cell function and vessel growth. Circ Res, 114: 1389-97. 24602777 10.1161/CIRCRESAHA.114.303265

[51] He C, Yang W, Yang J, Ding J, Li S, Wu H, et al (2017). Long Noncoding RNA MEG3 Negatively Regulates Proliferation and Angiogenesis in Vascular Endothelial Cells. DNA Cell Biol, 36: 475-481. 28418724 10.1089/dna.2017.3682

[52] Ruan W, Zhao F, Zhao S, Zhang L, Shi L, Pang T (2018). Knockdown of long noncoding RNA MEG3 impairs VEGF-stimulated endothelial sprouting angiogenesis via modulating VEGFR2 expression in human umbilical vein endothelial cells. Gene, 649: 32-39. 29391273 10.1016/j.gene.2018.01.072

[53] Nakamura K, Taguchi E, Miura T, Yamamoto A, Takahashi K, Bichat F, et al (2006). KRN951, a highly potent inhibitor of vascular endothelial growth factor receptor tyrosine kinases, has antitumor activities and affects functional vascular properties. Cancer Res, 66: 9134-42. 16982756 10.1158/0008-5472.CAN-05-4290

[54] Conigliaro A, Costa V, Lo Dico A, Saieva L, Buccheri S, Dieli F, et al (2015). CD90+ liver cancer cells modulate endothelial cell phenotype through the release of exosomes containing H19 lncRNA. Mol Cancer, 14: 155. 26272696 10.1186/s12943-015-0426-x PMC4536801

[55] Lang HL, Hu GW, Zhang B, Kuang W, Chen Y, Wu L, et al (2017). Glioma cells enhance angiogenesis and inhibit endothelial cell apoptosis through the release of exosomes that contain long non-coding RNA CCAT2. Oncol Rep, 38: 785-798. 28656228 10.3892/or.2017.5742 PMC5562059

[56] Kaneko S, Bonasio R, Saldana-Meyer R, Yoshida T, Son J, Nishino K, et al (2014). Interactions between JARID2 and noncoding RNAs regulate PRC2 recruitment to chromatin. Mol Cell, 53: 290-300. 24374312 10.1016/j.molcel.2013.11.012 PMC4026005

[57] Boon RA, Hofmann P, Michalik KM, Lozano-Vidal N, Berghauser D, Fischer A, et al (2016). Long Noncoding RNA Meg3 Controls Endothelial Cell Aging and Function: Implications for Regenerative Angiogenesis. J Am Coll Cardiol, 68: 2589-2591. 27931619 10.1016/j.jacc.2016.09.949

[58] Chen L, Yang W, Guo Y, Chen W, Zheng P, Zeng J, et al (2017). Exosomal lncRNA GAS5 regulates the apoptosis of macrophages and vascular endothelial cells in atherosclerosis. PLoS One, 12: e0185406. 28945793 10.1371/journal.pone.0185406 PMC5612752

[59] Gutschner T, Hammerle M, Diederichs S (2013). MALAT1 -- a paradigm for long noncoding RNA function in cancer. J Mol Med (Berl), 91: 791-801. 23529762 10.1007/s00109-013-1028-y

[60] Puthanveetil P, Chen S, Feng B, Gautam A, Chakrabarti S (2015). Long non-coding RNA MALAT1 regulates hyperglycaemia induced inflammatory process in the endothelial cells. J Cell Mol Med, 19: 1418-25. 25787249 10.1111/jcmm.12576 PMC4459855

[61] Maegdefessel L, Rayner KJ, Leeper NJ (2015). MicroRNA regulation of vascular smooth muscle function and phenotype: early career committee contribution. Arterioscler Thromb Vasc Biol, 35: 2-6. 25520518 10.1161/ATVBAHA.114.304877

[62] Robinson HC, Baker AH (2012). How do microRNAs affect vascular smooth muscle cell biology? Curr Opin Lipidol, 23: 405-11. 22964990 10.1097/MOL.0b013e32835719a1

[63] Liu X, Cheng Y, Chen X, Yang J, Xu L, Zhang C (2011). MicroRNA-31 regulated by the extracellular regulated kinase is involved in vascular smooth muscle cell growth via large tumor suppressor homolog 2. J Biol Chem, 286: 42371-80. 22020941 10.1074/jbc.M111.261065 PMC3234904

[64] Torella D, Iaconetti C, Catalucci D, Ellison GM, Leone A, Waring CD, et al (2011). MicroRNA-133 controls vascular smooth muscle cell phenotypic switch in vitro and vascular remodeling in vivo. Circ Res, 109: 880-93. 21852550 10.1161/CIRCRESAHA.111.240150

[65] Rangrez AY, M'Baya-Moutoula E, Metzinger-Le Meuth V, Henaut L, Djelouat MS, Benchitrit J, et al (2012). Inorganic phosphate accelerates the migration of vascular smooth muscle cells: evidence for the involvement of miR-223. PLoS One, 7: e47807. 23094093 10.1371/journal.pone.0047807 PMC3475714

[66] Yang X, Dong M, Wen H, Liu X, Zhang M, Ma L, et al (2017). MiR-26a contributes to the PDGF-BB-induced phenotypic switch of vascular smooth muscle cells by suppressing Smad1. Oncotarget, 8: 75844-75853. 29100273 10.18632/oncotarget.17998 PMC5652667

[67] Ji R, Cheng Y, Yue J, Yang J, Liu X, Chen H, et al (2007). MicroRNA expression signature and antisense-mediated depletion reveal an essential role of MicroRNA in vascular neointimal lesion formation. Circ Res, 100: 1579-88. 17478730 10.1161/CIRCRESAHA.106.141986

[68] Calvier L, Chouvarine P, Legchenko E, Hoffmann N, Geldner J, Borchert P, et al (2017). PPARgamma Links BMP2 and TGFbeta1 Pathways in Vascular Smooth Muscle Cells, Regulating Cell Proliferation and Glucose Metabolism. Cell Metab, 25: 1118-1134.e7. 28467929 10.1016/j.cmet.2017.03.011

[69] Liu X, Cheng Y, Zhang S, Lin Y, Yang J, Zhang C (2009). A necessary role of miR-221 and miR-222 in vascular smooth muscle cell proliferation and neointimal hyperplasia. Circ Res, 104: 476-87. 19150885 10.1161/CIRCRESAHA.108.185363 PMC2728290

[70] Davis BN, Hilyard AC, Nguyen PH, Lagna G, Hata A (2009). Induction of microRNA-221 by platelet-derived growth factor signaling is critical for modulation of vascular smooth muscle phenotype. J Biol Chem, 284: 3728-38. 19088079 10.1074/jbc.M808788200 PMC2635044

[71] Albinsson S, Sessa WC (2011). Can microRNAs control vascular smooth muscle phenotypic modulation and the response to injury? Physiol Genomics, 43: 529-33. 20841497 10.1152/physiolgenomics.00146.2010 PMC3110893

[72] Yu X, Li Z (2014). MicroRNAs regulate vascular smooth muscle cell functions in atherosclerosis (review). Int J Mol Med, 34: 923-33. 25197940 10.3892/ijmm.2014.1853

[73] Wang YS, Chou WW, Chen KC, Cheng HY, Lin RT, Juo SH (2012). MicroRNA-152 mediates DNMT1-regulated DNA methylation in the estrogen receptor alpha gene. PLoS One, 7: e30635. 22295098 10.1371/journal.pone.0030635 PMC3266286

[74] Ma X, Ma C, Zheng X (2013). MicroRNA-155 in the pathogenesis of atherosclerosis: a conflicting role? Heart Lung Circ, 22: 811-8. 23827206 10.1016/j.hlc.2013.05.651

[75] Tan M, Yan HB, Li JN, Li WK, Fu YY, Chen W, et al (2016). Thrombin Stimulated Platelet-Derived Exosomes Inhibit Platelet-Derived Growth Factor Receptor-Beta Expression in Vascular Smooth Muscle Cells. Cell Physiol Biochem, 38: 2348-65. 27198239 10.1159/000445588

[76] Salomon C, Yee S, Scholz-Romero K, Kobayashi M, Vaswani K, Kvaskoff D, et al (2014). Extravillous trophoblast cells-derived exosomes promote vascular smooth muscle cell migration. Front Pharmacol, 5: 175. 25157233 10.3389/fphar.2014.00175 PMC4128075

[77] Cho JR, Lee CY, Lee J, Seo HH, Choi E, Chung N, et al (2015). MicroRNA-761 inhibits Angiotensin II-induced vascular smooth muscle cell proliferation and migration by targeting mammalian target of rapamycin. Clin Hemorheol Microcirc, 63: 45-56. 26444612 10.3233/CH-151981

[78] Chen KC, Wang YS, Hu CY, Chang WC, Liao YC, Dai CY, et al (2011). OxLDL up-regulates microRNA-29b, leading to epigenetic modifications of MMP-2/MMP-9 genes: a novel mechanism for cardiovascular diseases. Faseb j, 25: 1718-28. 21266537 10.1096/fj.10-174904

[79] Deng L, Blanco FJ, Stevens H, Lu R, Caudrillier A, McBride M, et al (2015). MicroRNA-143 Activation Regulates Smooth Muscle and Endothelial Cell Crosstalk in Pulmonary Arterial Hypertension. Circ Res, 117: 870-883. 26311719 10.1161/CIRCRESAHA.115.306806 PMC4620852

[80] Gui T, Zhou G, Sun Y, Shimokado A, Itoh S, Oikawa K, et al (2012). MicroRNAs that target Ca(2+) transporters are involved in vascular smooth muscle cell calcification. Lab Invest, 92: 1250-9. 22688076 10.1038/labinvest.2012.85

[81] Kapustin AN, Chatrou ML, Drozdov I, Zheng Y, Davidson SM, Soong D, et al (2015). Vascular smooth muscle cell calcification is mediated by regulated exosome secretion. Circ Res, 116: 1312-23. 25711438 10.1161/CIRCRESAHA.116.305012

[82] Kapustin AN, Schoppet M, Schurgers LJ, Reynolds JL, McNair R, Heiss A, et al (2017). Prothrombin Loading of Vascular Smooth Muscle Cell-Derived Exosomes Regulates Coagulation and Calcification. Arterioscler Thromb Vasc Biol, 37: e22-e32. 28104608 10.1161/ATVBAHA.116.308886

[83] Du Y, Gao C, Liu Z, Wang L, Liu B, He F, et al (2012). Upregulation of a disintegrin and metalloproteinase with thrombospondin motifs-7 by miR-29 repression mediates vascular smooth muscle calcification. Arterioscler Thromb Vasc Biol, 32: 2580-8. 22995515 10.1161/ATVBAHA.112.300206

[84] Wen P, Cao H, Fang L, Ye H, Zhou Y, Jiang L, et al (2014). miR-125b/Ets1 axis regulates transdifferentiation and calcification of vascular smooth muscle cells in a high-phosphate environment. Exp Cell Res, 322: 302-12. 24486760 10.1016/j.yexcr.2014.01.025

[85] Goettsch C, Rauner M, Pacyna N, Hempel U, Bornstein SR, Hofbauer LC (2011). miR-125b regulates calcification of vascular smooth muscle cells. Am J Pathol, 179: 1594-600. 21806957 10.1016/j.ajpath.2011.06.016 PMC3181383

[86] Mathiyalagan P, Liang Y, Kim D, Misener S, Thorne T, Kamide CE, et al (2017). Angiogenic Mechanisms of Human CD34(+) Stem Cell Exosomes in the Repair of Ischemic Hindlimb. Circ Res, 120: 1466-1476. 28298297 10.1161/CIRCRESAHA.116.310557 PMC5420547

[87] Tan P, Wang YJ, Li S, Wang Y, He JY, Chen YY, et al (2016). The PI3K/Akt/mTOR pathway regulates the replicative senescence of human VSMCs. Mol Cell Biochem, 422: 1-10. 27619662 10.1007/s11010-016-2796-9

[88] Zhang L, Zhou M, Wang Y, Huang W, Qin G, Weintraub NL, et al (2014). miR-92a inhibits vascular smooth muscle cell apoptosis: role of the MKK4-JNK pathway. Apoptosis, 19: 975-83. 24705900 10.1007/s10495-014-0987-y PMC4143895

[89] Badi I, Burba I, Ruggeri C, Zeni F, Bertolotti M, Scopece A, et al (2015). MicroRNA-34a Induces Vascular Smooth Muscle Cells Senescence by SIRT1 Downregulation and Promotes the Expression of Age-Associated Pro-inflammatory Secretory Factors. J Gerontol A Biol Sci Med Sci, 70: 1304-11. 25352462 10.1093/gerona/glu180

[90] He JY, Tu C, Liu YS (2018). Role of lncRNA in aging and age-related diseases. Aging Medicine, 1: 158-75. 10.1002/agm2.12030 PMC6880696 31942494

[91] Wang Y, Song X, Li Z, Liu B (2018). Long non-coding RNAs in coronary atherosclerosis. Life Sci, 211: 189-197. 30195033 10.1016/j.lfs.2018.08.072

[92] Brock M, Schuoler C, Leuenberger C, Buhlmann C, Haider TJ, Vogel J, et al (2017). Analysis of hypoxia-induced noncoding RNAs reveals metastasis-associated lung adenocarcinoma transcript 1 as an important regulator of vascular smooth muscle cell proliferation. Exp Biol Med (Maywood), 242: 487-496. 28056547 10.1177/1535370216685434 PMC5367660

[93] Sun Z, Nie X, Sun S, Dong S, Yuan C, Li Y, et al (2017). Long Non-Coding RNA MEG3 Downregulation Triggers Human Pulmonary Artery Smooth Muscle Cell Proliferation and Migration via the p53 Signaling Pathway. Cell Physiol Biochem, 42: 2569-2581. 28848087 10.1159/000480218

[94] Wu G, Cai J, Han Y, Chen J, Huang ZP, Chen C, et al (2014). LincRNA-p21 regulates neointima formation, vascular smooth muscle cell proliferation, apoptosis, and atherosclerosis by enhancing p53 activity. Circulation, 130: 1452-1465. 25156994 10.1161/CIRCULATIONAHA.114.011675 PMC4244705

[95] Carrion K, Dyo J, Patel V, Sasik R, Mohamed SA, Hardiman G, et al (2014). The long non-coding HOTAIR is modulated by cyclic stretch and WNT/beta-CATENIN in human aortic valve cells and is a novel repressor of calcification genes. PLoS One, 9: e96577. 24788418 10.1371/journal.pone.0096577 PMC4006892

[96] Zhao L, Luo H, Li X, Li T, He J, Qi Q, et al (2017). Exosomes Derived from Human Pulmonary Artery Endothelial Cells Shift the Balance between Proliferation and Apoptosis of Smooth Muscle Cells. Cardiology, 137: 43-53. 28068653 10.1159/000453544

[97] Hergenreider E, Heydt S, Treguer K, Boettger T, Horrevoets AJ, Zeiher AM, et al (2012). Atheroprotective communication between endothelial cells and smooth muscle cells through miRNAs. Nat Cell Biol, 14: 249-56. 22327366 10.1038/ncb2441

[98] Cheng Y, Liu X, Yang J, Lin Y, Xu DZ, Lu Q, et al (2009). MicroRNA-145, a novel smooth muscle cell phenotypic marker and modulator, controls vascular neointimal lesion formation. Circ Res, 105: 158-66. 19542014 10.1161/CIRCRESAHA.109.197517 PMC2728297

[99] Chen NX, Kiattisunthorn K, O'Neill KD, Chen X, Moorthi RN, et al (2013). Decreased microRNA is involved in the vascular remodeling abnormalities in chronic kidney disease (CKD). PLoS One, 8: e64558. 23717629 10.1371/journal.pone.0064558 PMC3661525

[100] Lin X, He Y, Hou X, Zhang Z, Wang R, Wu Q (2016). Endothelial Cells Can Regulate Smooth Muscle Cells in Contractile Phenotype through the miR-206/ARF6&NCX1/Exosome Axis. PLoS One, 11: e0152959. 27031991 10.1371/journal.pone.0152959 PMC4816502

[101] Zheng B, Yin WN, Suzuki T, Zhang XH, Zhang Y, Song LL, et al (2017). Exosome-Mediated miR-155 Transfer from Smooth Muscle Cells to Endothelial Cells Induces Endothelial Injury and Promotes Atherosclerosis. Mol Ther, 25: 1279-1294. 28408180 10.1016/j.ymthe.2017.03.031 PMC5475247

[102] Pfeifer P, Werner N, Jansen F (2015). Role and Function of MicroRNAs in Extracellular Vesicles in Cardiovascular Biology. Biomed Res Int, 2015: 161393. 26558258 10.1155/2015/161393 PMC4618108

[103] Bazan HA, Hatfield SA, O'Malley CB, Brooks AJ, Lightell D Jr, Woods TC (2015). Acute Loss of miR-221 and miR-222 in the Atherosclerotic Plaque Shoulder Accompanies Plaque Rupture. Stroke, 46: 3285-7. 26451018 10.1161/STROKEAHA.115.010567 PMC4624519

[104] Fichtlscherer S, De Rosa S, Fox H, Schwietz T, Fischer A, Liebetrau C, et al (2010). Circulating microRNAs in patients with coronary artery disease. Circ Res, 107: 677-84. 20595655 10.1161/CIRCRESAHA.109.215566

[105] Mocharla P, Briand S, Giannotti G, Dorries C, Jakob P, Paneni F, et al .(2013). AngiomiR-126 expression and secretion from circulating CD34(+) and CD14(+) PBMCs: role for proangiogenic effects and alterations in type 2 diabetics. Blood, 121: 226-36. 23144172 10.1182/blood-2012-01-407106

[106] Jaiswal R, Luk F, Gong J, Mathys JM, Grau GE, Bebawy M (2012). Microparticle conferred microRNA profiles--implications in the transfer and dominance of cancer traits. Mol Cancer, 11: 37. 22682234 10.1186/1476-4598-11-37 PMC3499176

[107] Feld S, Kjellgren O, Smalling RW (1995). Aggressive interventional treatment of acute myocardial infarction. Lessons from the animal laboratory applied to the catheterization suite. Cardiology, 86: 365-73. 7585736 10.1159/000176903

[108] Feng Y, Huang W, Wani M, Yu X, Ashraf M (2014). Ischemic preconditioning potentiates the protective effect of stem cells through secretion of exosomes by targeting Mecp2 via miR-22. PLoS One, 9: e88685. 24558412 10.1371/journal.pone.0088685 PMC3928277

[109] De Rosa S, Fichtlscherer S, Lehmann R, Assmus B, Dimmeler S, Zeiher AM (2011). Transcoronary concentration gradients of circulating microRNAs. Circulation, 124: 1936-44. 21969012 10.1161/CIRCULATIONAHA.111.037572

[110] Zile MR, Mehurg SM, Arroyo JE, Stroud RE, DeSantis SM, Spinale FG (2011). Relationship between the temporal profile of plasma microRNA and left ventricular remodeling in patients after myocardial infarction. Circ Cardiovasc Genet, 4: 614-9. 21956146 10.1161/CIRCGENETICS.111.959841 PMC3535326

[111] Liew CC, Dzau VJ (2004). Molecular genetics and genomics of heart failure. Nat Rev Genet, 5: 811-25. 15520791 10.1038/nrg1470

[112] Melman YF, Shah R, Das S(2014). MicroRNAs in heart failure: is the picture becoming less miRky? Circ Heart Fail, 7: 203-14. 24449811 10.1161/CIRCHEARTFAILURE.113.000266

[113] Ikeda S, Pu WT (2010). Expression and function of microRNAs in heart disease. Curr Drug Targets, 11: 913-25. 20415651 10.2174/138945010791591304

[114] Matkovich SJ, Van Booven DJ, Youker KA, Torre-Amione G, Diwan A, Eschenbacher WH, et al (2009). Reciprocal regulation of myocardial microRNAs and messenger RNA in human cardiomyopathy and reversal of the microRNA signature by biomechanical support. Circulation, 119: 1263-71. 19237659 10.1161/CIRCULATIONAHA.108.813576 PMC2749457

[115] Naga Prasad SV, Duan ZH, Gupta MK, Surampudi VS, Volinia S, Calin GA, et al (2009). Unique microRNA profile in end-stage heart failure indicates alterations in specific cardiovascular signaling networks. J Biol Chem, 284: 27487-99. 19641226 10.1074/jbc.M109.036541 PMC2785678

[116] Wang M, Kim SH, Monticone RE, Lakatta EG (2015). Matrix metalloproteinases promote arterial remodeling in aging, hypertension, and atherosclerosis. Hypertension, 65: 698-703. 25667214 10.1161/HYPERTENSIONAHA.114.03618 PMC4359070

[117] Su SA, Xie Y, Fu Z, Wang Y, Wang JA, Xiang M (2017). Emerging role of exosome-mediated intercellular communication in vascular remodeling. Oncotarget, 8: 25700-25712. 28147325 10.18632/oncotarget.14878 PMC5421963

[118] Qi Y, Wang X, Rose KL, MacDonald WH, Zhang B, Schey KL, et al (2016). Activation of the Endogenous Renin-Angiotensin-Aldosterone System or Aldosterone Administration Increases Urinary Exosomal Sodium Channel Excretion. J Am Soc Nephrol, 27: 646-56. 26113616 10.1681/ASN.2014111137 PMC4731116

[119] Gildea JJ, Carlson JM, Schoeffel CD, Carey RM, Felder RA (2013). Urinary exosome miRNome analysis and its applications to salt sensitivity of blood pressure. Clin Biochem, 46: 1131-1134. 23726803 10.1016/j.clinbiochem.2013.05.052 PMC3786136

[120] Cunha PG, Boutouyrie P, Nilsson PM, Laurent S (2017). Early Vascular Ageing (EVA): Definitions and Clinical Applicability. Curr Hypertens Rev, 13: 8-15. 28412914 10.2174/1573402113666170413094319

[121] Blum A, Vaispapir V, Keinan-Boker L, Soboh S, Yehuda H, Tamir S (2012). Endothelial dysfunction and procoagulant activity in acute ischemic stroke. J Vasc Interv Neurol, 5: 33-9. 22737264 PMC3379907

[122] Geng W, Tang H, Luo S, Lv Y, Liang D, Kang X, et al (2019). Exosomes from miRNA-126-modified ADSCs promotes functional recovery after stroke in rats by improving neurogenesis and suppressing microglia activation. Am J Transl Res, 11: 780-792. 30899379 PMC6413259

[123] Jiang M, Wang H, Jin M, Yang X, Ji H, Jiang Y, et al (2018). Exosomes from MiR-30d-5p-ADSCs Reverse Acute Ischemic Stroke-Induced, Autophagy-Mediated Brain Injury by Promoting M2 Microglial/Macrophage Polarization. Cell Physiol Biochem, 47: 864-878. 29807362 10.1159/000490078

[124] Kubota K, Nakano M, Kobayashi E, Mizue Y, Chikenji T, Otani M, et al (2018). An enriched environment prevents diabetes-induced cognitive impairment in rats by enhancing exosomal miR-146a secretion from endogenous bone marrow-derived mesenchymal stem cells. PLoS One, 13: e0204252. 30240403 10.1371/journal.pone.0204252 PMC6150479

[125] Safar ME, London GM, Plante GE (2004). Arterial stiffness and kidney function. Hypertension, 43: 163-8. 14732732 10.1161/01.HYP.0000114571.75762.b0

[126] Yu Y, Bai F, Qin N, Liu W, Sun Q, Zhou Y, et al (2018). Non-Proximal Renal Tubule-Derived Urinary Exosomal miR-200b as a Biomarker of Renal Fibrosis. Nephron, 139: 269-282. 29539618 10.1159/000487104

[127] Lange T, Stracke S, Rettig R, Lendeckel U, Kuhn J, Schluter R, et al (2017). Identification of miR-16 as an endogenous reference gene for the normalization of urinary exosomal miRNA expression data from CKD patients. PLoS One, 12: e0183435. 28859135 10.1371/journal.pone.0183435 PMC5578666

[128] Vogiatzi G, Oikonomou E, Deftereos S, Siasos G, Tousoulis D (2018). Peripheral artery disease: a micro-RNA-related condition? Curr Opin Pharmacol, 39: 105-112. 29679926 10.1016/j.coph.2018.04.001

[129] Bonauer A, Carmona G, Iwasaki M, Mione M, Koyanagi M, Fischer A, et al (2009). MicroRNA-92a controls angiogenesis and functional recovery of ischemic tissues in mice. Science, 324: 1710-3. 19460962 10.1126/science.1174381

[130] Matsumoto S, Sakata Y, Suna S, Nakatani D, Usami M, Hara M, et al (2013). Circulating p53-responsive microRNAs are predictive indicators of heart failure after acute myocardial infarction. Circ Res, 113: 322-6. 23743335 10.1161/CIRCRESAHA.113.301209

[131] Geriatric Cardiology Group of Chinese Geriatric Society(2018). Expert consensus on clinical assessment and intervention of vascular aging in China. Chin J Geriatr, 37: 1177-1184.

[132] D'Agostino RB Sr, Vasan RS, Pencina MJ, Wolf PA, Cobain M, Massaro JM, et al (2008). General cardiovascular risk profile for use in primary care: the Framingham Heart Study. Circulation, 117: 743-53. 18212285 10.1161/CIRCULATIONAHA.107.699579

[133] Khoshdel AR, Thakkinstian A, Carney SL, Attia J (2006). Estimation of an age-specific reference interval for pulse wave velocity: a meta-analysis. J Hypertens, 24: 1231-7. 16794467 10.1097/01.hjh.0000234098.85497.31

[134] Li SS, Zhang CT, Zhou HL, Huang K, Fsn QP (2012). Correlation of brachial ankle pulse wave velocity with cardiovascular risks and Framingham score. Chin J Mult Organ Dis Elderly, 11: 912-916.

[135] Xu Y, Arora RC, Hiebert BM, Lerner B, Szwajcer A, McDonald K, et al (2014). Non-invasive endothelial function testing and the risk of adverse outcomes: a systematic review and meta-analysis. Eur Heart J Cardiovasc Imaging, 15: 736-46. 24399339 10.1093/ehjci/jet256

[136] Celermajer DS, Sorensen KE, Spiegelhalter DJ, Georgakopoulos D, Robinson J, Deanfield JE (1994). Aging is associated with endothelial dysfunction in healthy men years before the age-related decline in women. J Am Coll Cardiol, 24: 471-6. 8034885 10.1016/0735-1097(94)90305-0

[137] Wang Y, Tao J, Yang Z, Tu C, Xu MG, Wang JM, et al (2005). Tumor necrosis factor-α induces release of endothelial microparticle from endothelial cells. Chin J Cardiol, 32: 1137-1140. 16563288

[138] Xia WH, Li J, Su C, Yang Z, Chen L, Wu F, et al (2012). Physical exercise attenuates age-associated reduction in endothelium-reparative capacity of endothelial progenitor cells by increasing CXCR4/JAK-2 signaling in healthy men. Aging Cell, 11: 111-9. 22018014 10.1111/j.1474-9726.2011.00758.x

[139] Tao J, Wang Y, Yang Z, Tu C, Xu MG, Wang JM (2006). Circulating endothelial progenitor cell deficiency contributes to impaired arterial elasticity in persons of advancing age. J Hum Hypertens, 20: 490-5. 16496018 10.1038/sj.jhh.1001996

[140] Xitong D, Xiaorong Z (2016). Targeted therapeutic delivery using engineered exosomes and its applications in cardiovascular diseases. Gene, 575: 377-384. 26341056 10.1016/j.gene.2015.08.067

[141] Boukouris S, Mathivanan S (2015). Exosomes in bodily fluids are a highly stable resource of disease biomarkers. Proteomics Clin Appl, 9: 358-67. 25684126 10.1002/prca.201400114 PMC5502131

